# Metabolic engineering of *Bacillus amyloliquefaciens* for enhanced production of *S*-adenosylmethionine by coupling of an engineered *S*-adenosylmethionine pathway and the tricarboxylic acid cycle

**DOI:** 10.1186/s13068-019-1554-0

**Published:** 2019-09-09

**Authors:** Liying Ruan, Lu Li, Dian Zou, Cong Jiang, Zhiyou Wen, Shouwen Chen, Yu Deng, Xuetuan Wei

**Affiliations:** 1 Key Laboratory of Environment Correlative Dietology (Ministry of Education), College of Food Science and Technology, Huazhong Agricultural University, Wuhan, 430070 China; 2 National Engineering Laboratory for Cereal Fermentation Technology (NELCF), Jiangnan University, Wuxi, 214122 China; 3 Hubei Collaborative Innovation Center for Green Transformation of Bio-Resources, College of Life Sciences, Hubei University, Wuhan, 430062 China; 4 Department of Food Science and Human Nutrition, Iowa State University, Ames, 50011 USA

**Keywords:** *S*-Adenosylmethionine, Pathway engineering, Pathway coupling, *Bacillus amyloliquefaciens*

## Abstract

**Background:**

*S*-Adenosylmethionine (SAM) is a critical cofactor involved in many biochemical reactions. However, the low fermentation titer of SAM in methionine-free medium hampers commercial-scale production. The SAM synthesis pathway is specially related to the tricarboxylic acid (TCA) cycle in *Bacillus amyloliquefaciens*. Therefore, the SAM synthesis pathway was engineered and coupled with the TCA cycle in *B. amyloliquefaciens* to improve SAM production in methionine-free medium.

**Results:**

Four genes were found to significantly affect SAM production, including *SAM2* from *Saccharomyces cerevisiae*, *metA* and *metB* from *Escherichia coli*, and native *mccA*. These four genes were combined to engineer the SAM pathway, resulting in a 1.42-fold increase in SAM titer using recombinant strain HSAM1. The engineered SAM pathway was subsequently coupled with the TCA cycle through deletion of succinyl-CoA synthetase gene *sucC*, and the resulted HSAM2 mutant produced a maximum SAM titer of 107.47 mg/L, representing a 0.59-fold increase over HSAM1. Expression of *SAM2* in this strain via a recombinant plasmid resulted in strain HSAM3 that produced 648.99 mg/L SAM following semi-continuous flask batch fermentation, a much higher yield than previously reported for methionine-free medium.

**Conclusions:**

This study reports an efficient strategy for improving SAM production that can also be applied for generation of SAM cofactors supporting group transfer reactions, which could benefit metabolic engineering, chemical biology and synthetic biology.

## Background

*S*-Adenosylmethionine (SAM) is one of the most widely used cofactors for group transfer reactions involved in various metabolic processes, and it serves as the main methyl donor for methylation of DNA, proteins and secondary metabolites, as well as 5-deoxyadenosyl radical, 1-aminopropyl, and 2-aminobutyryl donors [1, 2]. SAM-dependent group transfer reactions can be broadly applied in the fields of chemical biology, synthetic biology and metabolic engineering [3–5]. In particular, SAM-dependent methylation is the critical step for production of pharmaceuticals and fine chemicals [3, 4]. Moreover, SAM has multiple beneficial effects for human health and has been used as a functional nutriment or drug for the prevention and treatment of liver disease, osteoarthritis and depression [6, 7]. SAM also has the potential for extending lifespan and treating colon cancer [8, 9]. Given the critical functions and market demand, SAM is attracting much interest.

SAM can be synthesized by SAM synthetase from methionine and ATP [7, 10]. Low availability of the precursor methionine is considered as the limiting factor for SAM production [11–13]. Consequently, methionine is usually added directly into the medium as a fermentation substrate to produce SAM and various previous studies have aimed to improve SAM production from methionine, including fermentation optimization, conventional mutation breeding, and genetic engineering [7, 14]. The conversion rate from expensive methionine to SAM ranges from 15 to 42% [15–17], resulting in high cost. Therefore, development of efficient SAM production methods using methionine-free medium is much needed.

**Fig. 1:**
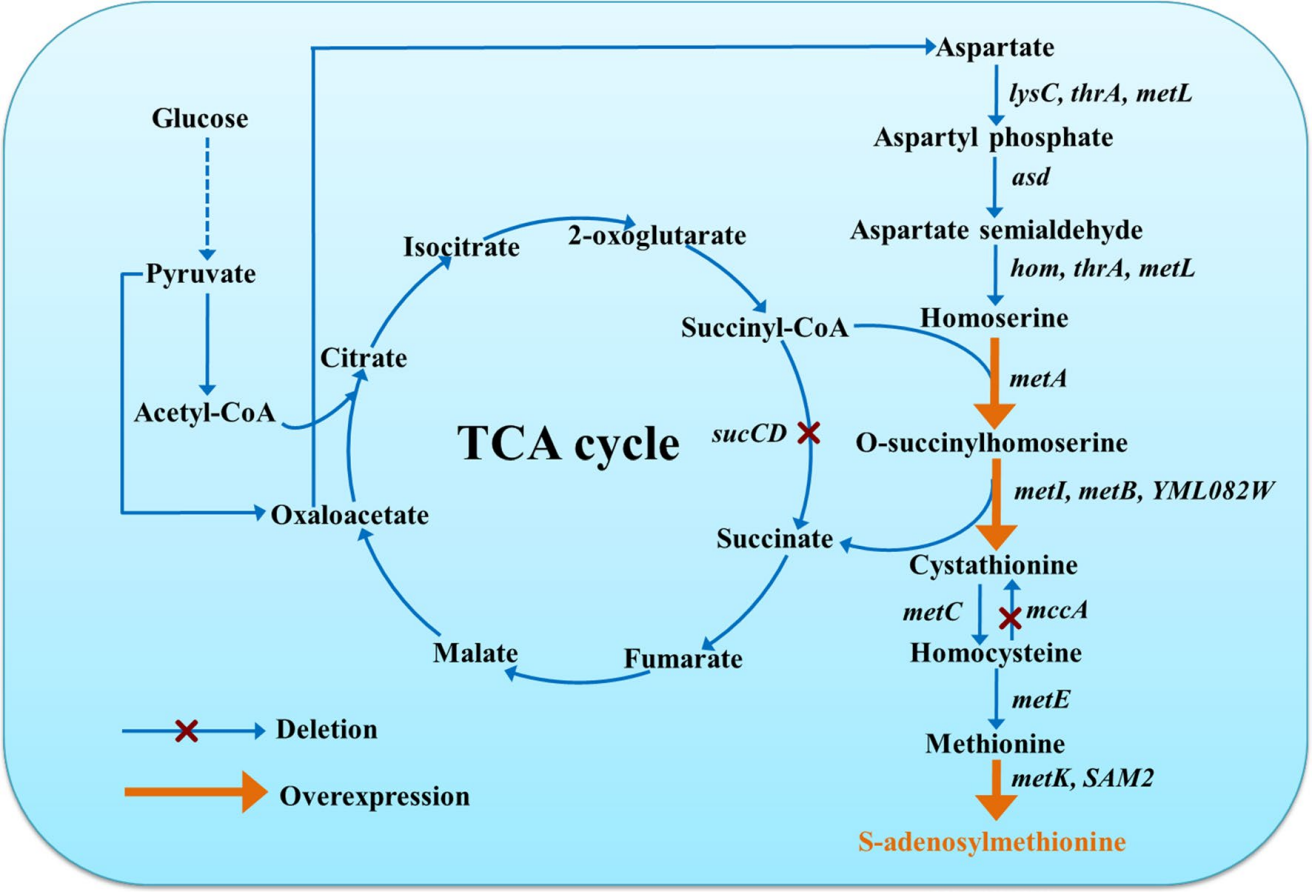
Metabolic engineering strategies for coupling the engineered SAM synthesis pathway and the TCA cycle in *Bacillus amyloliquefaciens*. Genes are as follows: aspartokinase (encoded by *lysC*), bifunctional aspartokinase/homoserine dehydrogenase (*thrA* or *metL*), aspartate-semialdehyde dehydrogenase (*asd*), homoserine dehydrogenase (*hom*), homoserine *O*-succinyltransferase (*metA*), cystathionine-γ-synthase (*metI*, *metB* or *YML082W*), cystathionine-β-lyase (*metC*), cystathionine-β-synthase (*mccA*), methionine synthase (*metE*), SAM synthetase (*metK* or *SAM2*), succinyl-CoA synthetase (*sucCD*)

Researchers have attempted to manipulate the SAM biosynthesis pathway to produce SAM using low-cost carbohydrates or aspartate as substrates [13, 15, 18]. In this pathway, aspartate is synthesized from glucose via the glycolytic pathway and the tricarboxylic acid (TCA) cycle and then converted to SAM through the SAM synthesis pathway (Fig. 1), which includes aspartokinase (encoded by *lysC*), bifunctional aspartokinase/homoserine dehydrogenase (*thrA* or *metL*), aspartate-semialdehyde dehydrogenase (*asd*), homoserine dehydrogenase (*hom*), homoserine O-succinyltransferase (*metA*), cystathionine-γ-synthase (*metI*, *metB* or *YML082W*), cystathionine-β-lyase (*metC*), cystathionine-β-synthase (*mccA*), methionine synthase (*metE*) and SAM synthetase (*metK* or *SAM2*) [15, 18]. In *Escherichia coli*, the SAM titer was improved in methionine-free medium via regulation of NADPH and ATP, but remained below 10 mg/L [13, 18]. In *Corynebacterium glutamicum*, four genes (*mcbR*, *thrB*, *metB* and *Ncgl2640*) were deleted and two genes (*vgb* and *metK*) were overexpressed, resulting in a maximum SAM titer of 196.7 mg/L in methionine-free medium [15]. Although the SAM titer was improved in methionine-free medium, it is still not high enough from a commercial production perspective. Therefore, effort is needed to fully explore the production potential using low-cost substrates.

Coupling to the TCA cycle can be effective for improving the production of lysine and 7-aminodeacetoxycephalosporanic acid [19, 20]. In the SAM synthesis pathway of some bacteria (Fig. 1), succinyl-CoA serves as the cosubstrate with homoserine to synthesize *O*-succinylhomoserine, which is then converted to cystathionine and succinate. On the other hand, succinyl-CoA can be converted to succinate by succinyl-CoA synthetase in the TCA cycle [19, 20]. Thus, the TCA cycle competes for succinyl-CoA with the SAM pathway. If the native TCA cycle is blocked by disrupting succinyl-CoA synthetase, more succinyl-CoA may be driven to flux into the SAM pathway to promote SAM production. If the SAM pathway is strong enough, it could generate abundant succinate to compensate for the blocked TCA cycle, supporting recovery of cell growth. The SAM pathway and the TCA cycle could, therefore, be coupled to enhance SAM production collaboratively, but this has not yet been demonstrated in practice.

*Bacillus* species, especially *Bacillus subtilis*, *Bacillus amyloliquefaciens* and *Bacillus licheniformis*, have been widely applied in the fields of medicine, food, cosmetics and agriculture [21, 22]. Due to the rapid growth, high robustness and rich genetic tools, *Bacillus* species have been engineered as industrial workhorses for production of various products [23], such as engineering *B. subtilis* to synthesize riboflavin, pyridoxine, *N*-acetylglucosamine and hyaluronan [24–27], and engineering *B. amyloliquefaciens* to produce poly-γ-glutamic acid, levan and menaquinone-7 [28–30]. *B. amyloliquefaciens* is a promising chassis cell and related metabolic engineering tools have been developed. Moreover, *B. amyloliquefaciens* deficient in tetrahydropicolinate succinylase cannot mediate succinyl-CoA into the lysine pathway, while it may just drive succinyl-CoA into the SAM synthesis pathway by homoserine *O*-succinyltransferase. Therefore, *B. amyloliquefaciens* have the potential to couple the SAM synthesis pathway with the TCA cycle to enhance SAM production. The present study aimed to enhance SAM production using *B. amyloliquefaciens* HZ-12 in methionine-free medium by coupling a strong SAM synthesis pathway with the TCA cycle. Associated genes were engineered systematically to strengthen the SAM pathway and succinyl-CoA synthetase was disrupted to force succinyl-CoA flux through the engineered SAM pathway to enter the TCA cycle, thereby coupling the engineered SAM pathway and the TCA cycle (Fig. 1).

## Results and discussion

### Effects of key genes on SAM production

**Fig. 2:**
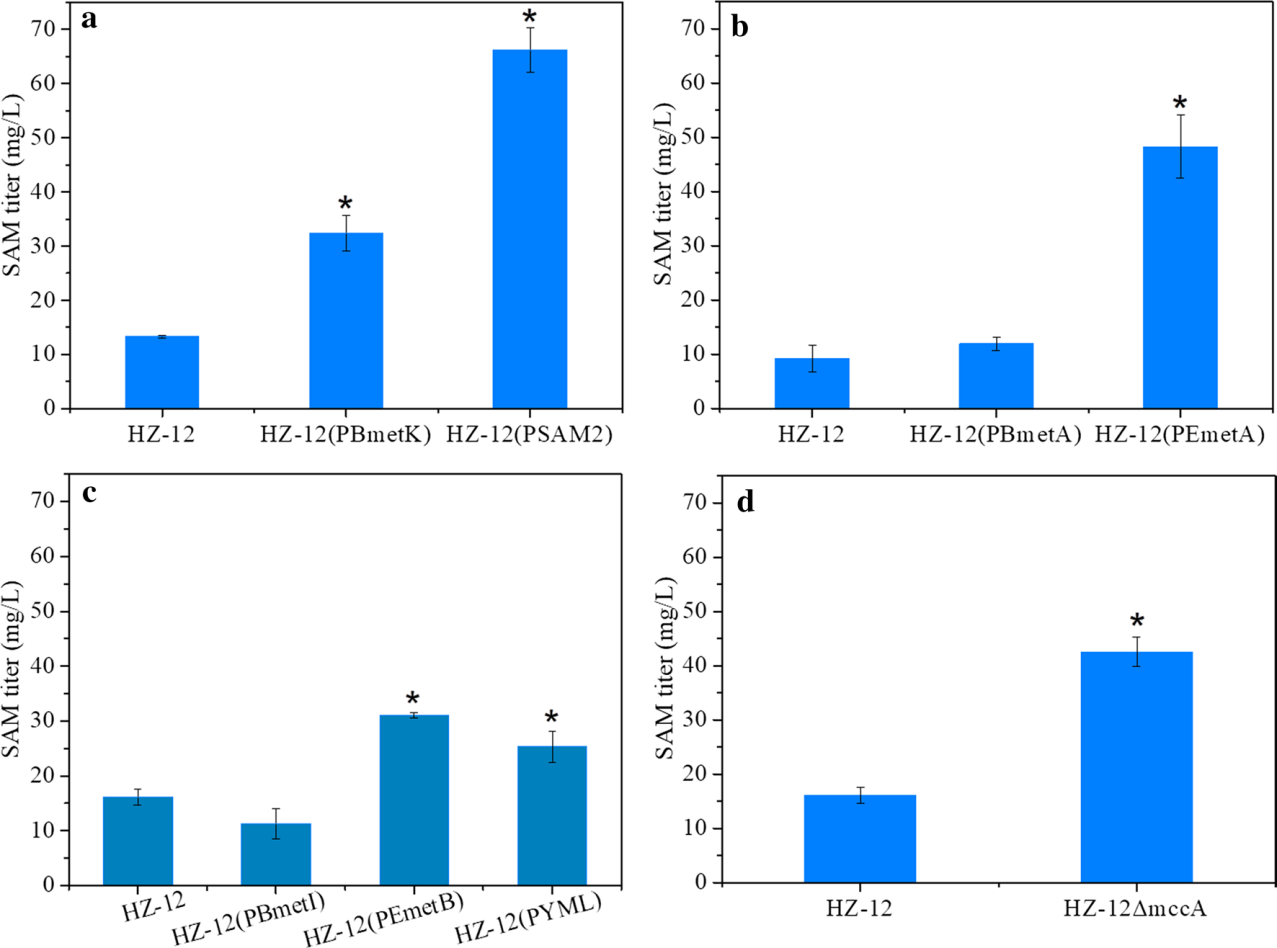
Effects of key genes on SAM production. **a** HZ-12 (PBmetK) expressing *metK* from *B. amyloliquefaciens* and HZ-12 (PSAM2) expressing *SAM2* from *S. cerevisiae.* **b** HZ-12 (PBmetA) expressing *metA* from *B. amyloliquefaciens* and HZ-12 (PEmetA) expressing *metA* from *E. coli*. **c** HZ-12 (PBmetI) expressing *metI* from *B. amyloliquefaciens*, HZ-12 (PEmetB) expressing *metB* from *E. coli*, and HZ-12 (PYML) expressing *YML082* *W* from *S. cerevisiae*. **d** HZ-12Δ*mccA* carrying deleted *mccA*. All data were obtained under the initial fermentation medium. Data are expressed as mean ± standard deviation (SD) from triplicate measurements. Asterisks indicate a significant difference (*p* < 0.05) compared with the control strain (HZ-12)

SAM synthetase is a critical enzyme that catalyzes SAM synthesis from methionine and ATP, and overexpression of the genes involved can enhance SAM production, especially the *SAM2* gene from *S. cerevisiae* [14, 31]. However, whether *B. amyloliquefaciens* SAM synthetase gene *metK* affects SAM production has not been investigated. Therefore, *metK* from *B. amyloliquefaciens* and *SAM2* from *S. cerevisiae* were expressed in HZ-12, resulting in recombinant strains HZ-12 (PBmetK) and HZ-12 (PSAM2), respectively. These recombinant strains were fermented for 42 h in initial fermented medium to compare SAM titers. As shown in Fig. 2a, expression of *metK* and *SAM2* significantly improved SAM titers, and the maximum SAM titer (66.22 mg/L) was achieved by HZ-12 (PSAM2). These results indicate that *SAM2* is the preferred target gene for enhancement of SAM production, similar to the results of other studies [7, 31].

Homoserine *O*-succinyltransferase (encoded by *metA*) combines homoserine and succinyl-CoA to synthesize *O*-succinylhomoserine [32]. This step is critical for driving succinyl-CoA into the SAM pathway, hence strengthening the expression of *metA* is likely to increase competition for succinyl-CoA for SAM synthesis. In *E. coli*, *metA* can efficiently enhance the production of methionine, the precursor of SAM [33, 34]. In the present work, *metA* genes from *E. coli* and *B. amyloliquefaciens* were expressed and compared (Fig. 2b). In HZ12 (PEmetA) harboring *metA* from *E. coli*, the SAM titer reached 48.28 mg/L after 42 h in initial fermentation medium, higher than that of wild-type strain HZ-12, whereas overexpression of native *metA* in HZ-12 yielded no significant enhancement. These results confirmed that *metA* from *E. coli* is crucial for enhancing SAM production by *B. amyloliquefaciens*.

Cystathionine-γ-synthase catalyzes the reaction between O-succinylhomoserine and cysteine to produce cystathionine and succinate, a key step in the generation of succinate from the SAM pathway. Overexpression of the cystathionine-γ-synthase gene can also significantly improve methionine production in *E. coli* [33]. In the present work, cystathionine-γ-synthase genes from HZ-12 (*metI*), *E. coli* (*metB*) and *S. cerevisiae* (*YML082W*) were expressed, resulting in recombinant strains HZ-12 (PBmetI), HZ-12 (PEmetB) and HZ-12 (PYML), respectively. As shown in Fig. 2c, expression of *metB* and *YML082* *W* markedly improved SAM titers in initial fermentation medium, while no significant change was observed for the *metI*-expressing strain. Since strain HZ-12 (PEmetB) produced the maximum SAM titer of 31.06 mg/L, *metB* from *E. coli* was chosen for further investigation.

Cystathionine-β-synthase catalyzes the conversion of homocysteine to cystathionine, a reverse reaction in the SAM synthesis pathway, and knockout of the cystathionine-β-synthase gene can improve SAM production in *Pichia pastoris* [35]. Therefore, we deleted the *mccA* gene encoding cystathionine-β-synthase in HZ-12, and the SAM titer reached 42.56 mg/L in HZ-12Δ*mccA* in initial fermentation medium, much higher than that of HZ-12 (Fig. 2d). In addition, we also manipulated other genes in the SAM synthesis pathway, including expression of bifunctional aspartokinase/homoserine dehydrogenase genes (*thrA* and *metL*) [36], and deletion of the anti-antiterminator of the *metE* gene in HZ-12 [37]. However, expression of *thrA* and *metL* significantly reduced SAM production (Additional file 1: Fig. S1), and the SAM titer was only slightly improved after deletion of the anti-antiterminator of the *metE* gene (Additional file 1: Fig. S2). Various genes influencing SAM synthesis were identified, including *SAM2* from *S. cerevisiae*, *metA* and *metB* from *E. coli*, as well as the native *mccA*, among which *SAM2* had the strongest effect on SAM synthesis.

### Combined effects of beneficial SAM synthesis pathway genes

**Fig. 3:**
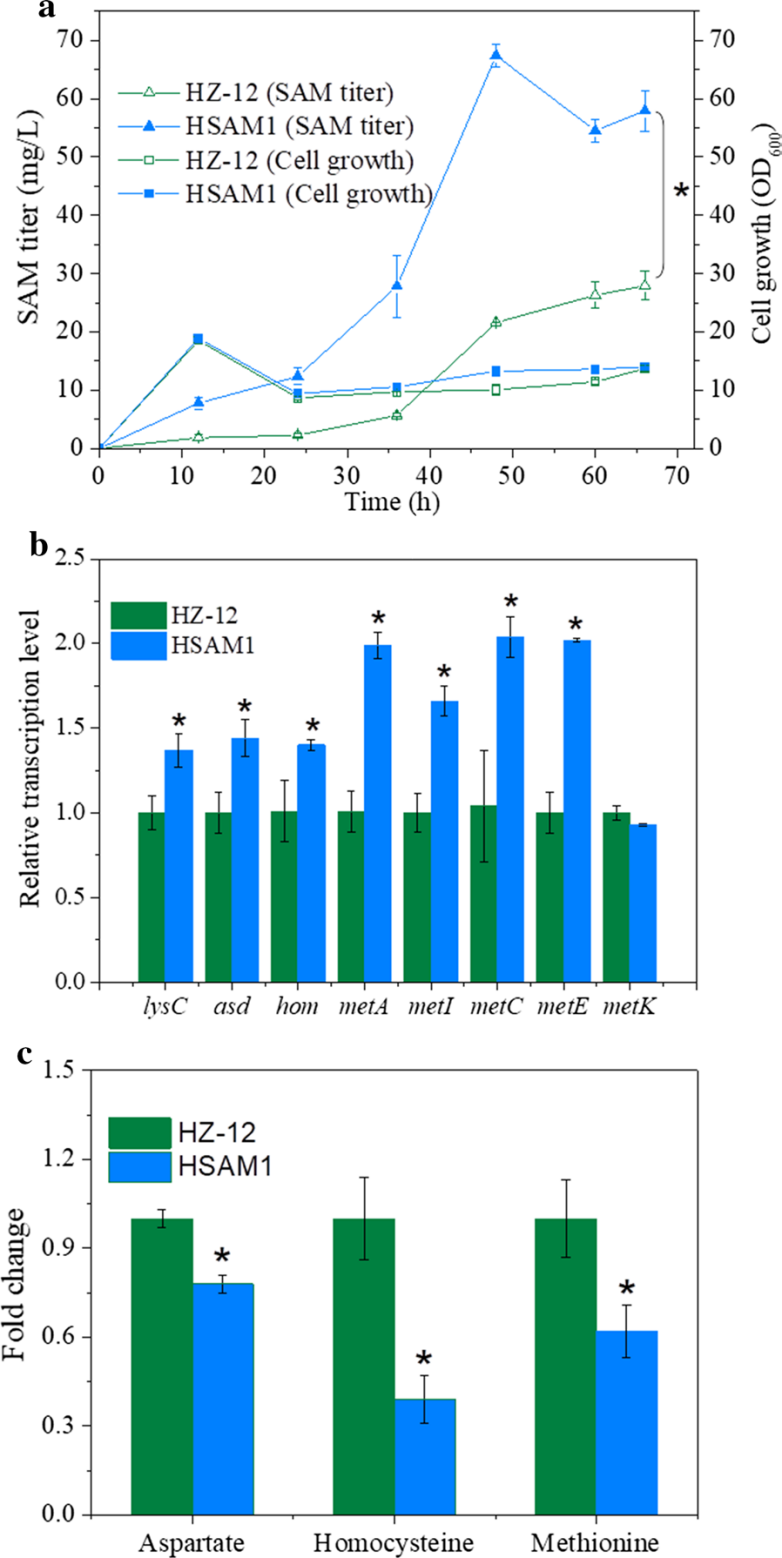
Effects of combining beneficial genes on SAM synthesis. **a** SAM titer and cell growth. **b** Relative transcription levels of SAM synthesis pathway-related genes. **c** Fold change of key metabolites. All data were obtained under the initial fermentation medium. Data are expressed as mean ± SD from triplicate measurements. Asterisks indicate a significant difference (*p* < 0.05) between HZ-12 and HSAM1

All the aforementioned beneficial genetic manipulations were combined in a single strain. Specifically, *SAM2* from *S. cerevisiae* and *metA* and *metB* from *E. coli* were integrated into the genome of HZ-12, and the *mccA* gene was deleted, resulting in strain HSAM1. Fermentation profiles of strains HZ-12 and HSAM1 were compared in initial fermentation medium. As shown in Fig. 3a, the SAM titer of HSAM1 increased with the increasing fermentation time, and the maximum titer reached 67.41 mg/L at 48 h, 1.42-fold higher than that of HZ-12 (27.91 mg/mL). The cell growth trend of HSAM1 was similar to that of HZ-12 (Fig. 3a). The above results showed that the SAM titer of strain HSAM1 was not significantly improved over that of strain HZ-12 harboring plasmid PSAM2. This result was probably due to the low gene copy number during integration expression. Each HSAM1 cell carries only a single copy of the integrated *SAM2*, *metA* and *metB* genes, whereas there are 5–20 copies of the *SAM2* gene in each HZ-12 (PSAM2) cell due to the high copy number of the pHY300PLK-based plasmid [38].

Transcription levels of native SAM synthesis pathway genes between HSAM1 and HZ-12 were also compared. As shown in Fig. 3b, transcription levels of most native SAM synthesis genes in strain HSAM1 were higher than those in HZ-12, with ~ twofold improvement observed for *metA*, *metC* and *metE*. These results indicate that combining genetic manipulations enhances the overall SAM synthesis pathway. The transcription level of native *metK* gene showed no significant difference, probably because the integrated *SAM2* gene was strong enough to mediate the SAM synthesis. Changes in the key intracellular metabolites of the SAM synthesis pathway were also measured. In HSAM1, the abundance of aspartate and homocysteine, located in the upstream pathway of modified genes, was 22% and 61% lower, respectively, than in HZ-12 (Fig. 3c), indicating that combined downstream genetic manipulations affected upstream substrate consumption. As expected for the intermediate metabolite of the modified gene products, methionine was also decreased significantly (Fig. 3c), probably because the *SAM2* gene is more efficient than *metA*, *metB* and *mccA*. The greater substrate consumption further confirmed that the combined genetic manipulations drove metabolic flux for improved SAM synthesis.

### Coupling the engineered SAM synthesis pathway with the TCA cycle

**Fig. 4:**
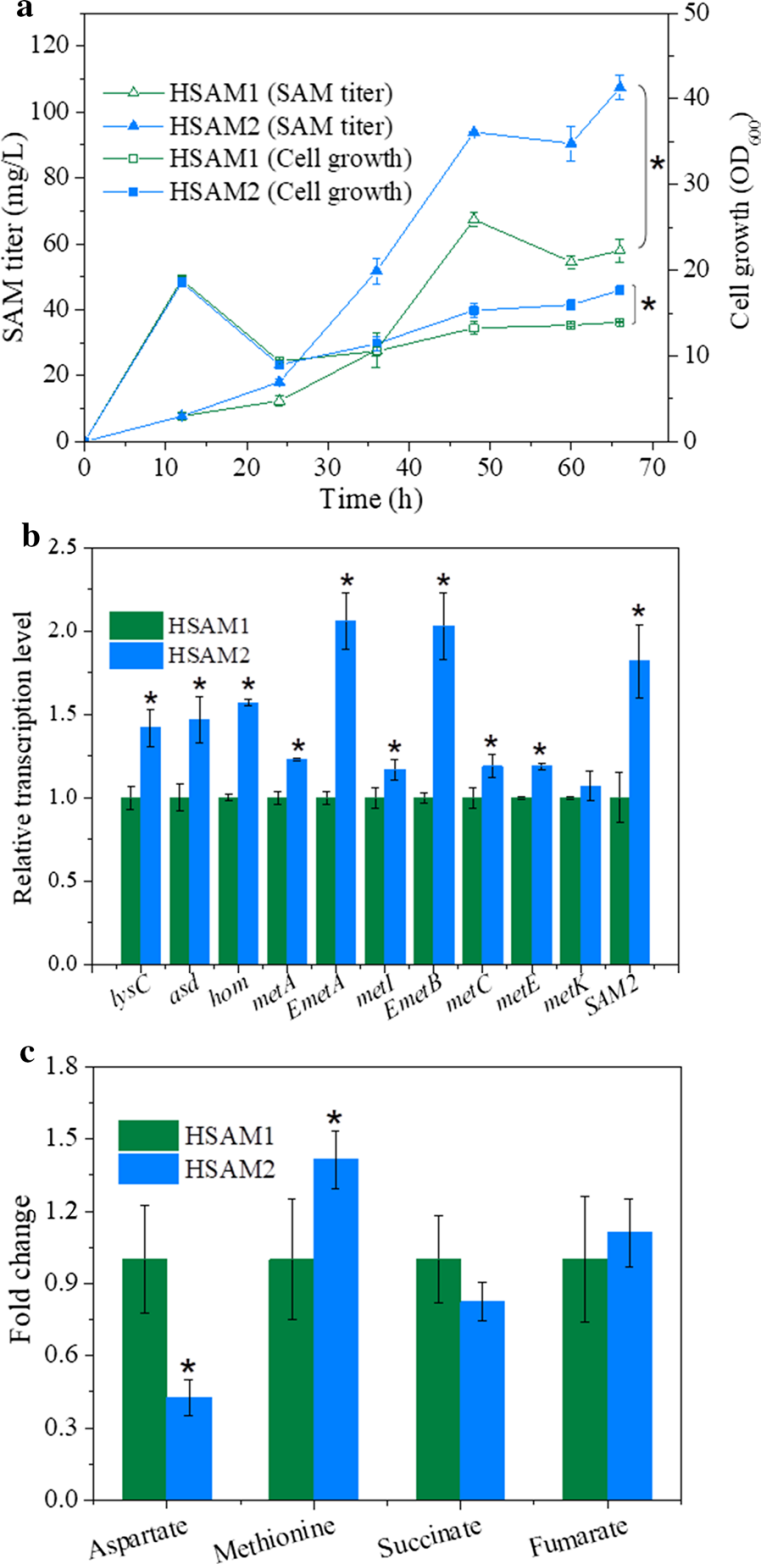
Effects of pathway coupling on SAM synthesis. **a** SAM titer and cell growth. **b** Relative transcription levels of SAM synthesis pathway-related genes. **c** Fold change of key metabolites. All data were obtained under the initial fermentation medium. Data are expressed as mean ± SD from triplicate measurements. Asterisks indicate a significant difference (*p* < 0.05) between HSAM1 and HSAM2

The biosynthesis of SAM requires the cosubstrate succinyl-CoA, which is also a key intermediate of the TCA cycle [19, 20]. The TCA cycle competes with the SAM synthesis pathway for succinyl-CoA. If the succinyl-CoA synthetase gene is deleted to block succinyl-CoA consumption by the TCA cycle, the SAM pathway would likely receive more succinyl-CoA, thereby enhancing SAM synthesis. Moreover, the improved SAM pathway would be expected to release more succinate to compensate for the TCA cycle and cell growth. Thus, the SAM pathway would presumably couple with the TCA cycle to coordinate SAM production and cell growth. To verify this hypothesis, succinyl-CoA synthetase was inactivated by deleting the *sucC* gene from HSAM1, resulting in strain HSAM2. As shown in Fig. 4a, the SAM titer of HSAM2 reached 107.47 mg/L in the initial fermentation medium, representing a 0.59-fold increase over that of HSAM1.

Transcription analysis was also performed to investigate the effect of *sucC* deletion on the SAM synthesis pathway. Compared with the control strain, transcription levels of most SAM pathway-associated genes were increased (Fig. 4b), especially the integrated *SAM2* gene, and *metA* and *metB* from *E. coli* (designated as *EmetA* and *EmetA*), all elevated ~ twofold, which were much higher than that of corresponding native genes (*metA*, *metI* and *metK*) of *B. amyloliquefaciens*. According to Fig. 1, genes of *EmetA*, *EmetB* and *SAM2* were more effective than corresponding native genes in *B. amyloliquefaciens* to improve SAM production, suggesting that those three integrated genes were more efficient to affect the SAM synthesis pathway. Bacteria preferably utilized efficient genes and enzymes to cope with stress perturbations. Therefore, transcriptional levels of these three integrated genes were improved more obviously than that of wild-type genes after deletion of *sucC*. These results confirmed that deletion of *sucC* forced more succinyl-CoA through the SAM synthesis pathway. GC–MS analysis showed that the medium substrate aspartate was reduced by 57% after knockout of *sucC*, and the downstream metabolite methionine was increased by 41% (Fig. 4c), further confirming that the SAM synthesis pathway was enhanced. These results indicate that our pathway coupling strategy could also be applied to enhance methionine production.

Conversely, no significant concentration change was observed for intracellular succinate or fumarate intermediates of the TCA cycle (Fig. 4c), and cell growth did not decline after deletion of *sucC* (Fig. 4a), suggesting that succinate generated from the engineered SAM pathway likely compensates for the TCA cycle and cell growth. In *C. glutamicum*, succinyl-CoA synthetase was mutated to couple the TCA cycle and the lysine synthesis pathway, leading to a 0.60-fold increase in lysine yield, and succinate generated from the lysine pathway and the glyoxylate shunt was believed to compensate for the TCA cycle and cell growth in *C. glutamicum* [19]. *Bacillus* species usually utilize acetyl-CoA as cosubstrate for lysine synthesis instead of succinyl-CoA [39]. KEGG database analysis showed that, unlike *C. glutamicum*, *B. amyloliquefaciens* lacks tetrahydrodipicolinate succinylase, the enzyme responsible for feeding succinyl-CoA into the lysine pathway. According to the KEGG database, a glyoxylate shunt is not present in *B. amyloliquefaciens*, and the metabolite glyoxylate was not detected in our GC–MS analysis. Therefore, succinate generated from the engineered SAM pathway was believed to compensate for the TCA cycle and cell growth after deletion of *sucC* from HSAM1. We also compared cell growth between the wild-type strain (HZ-12), the *sucC*-deficient strain (HZ-12ΔsucC) and the *sucC*-deficient strain harboring the engineered SAM pathway (HSAM2). As shown in Additional file 1: Fig. S3, cell growth of HZ-12ΔsucC was much lower than HZ-12, indicating that the native SAM pathway could not recover cell growth completely after deletion of *sucC*. Interestingly, HSAM2 showed no significant difference from HZ-12, confirming that the engineered SAM pathway could compensate for the TCA cycle to maintain normal cell growth in the *sucC*-deficient strain.

### SAM synthesis from HSAM3

**Fig. 5:**
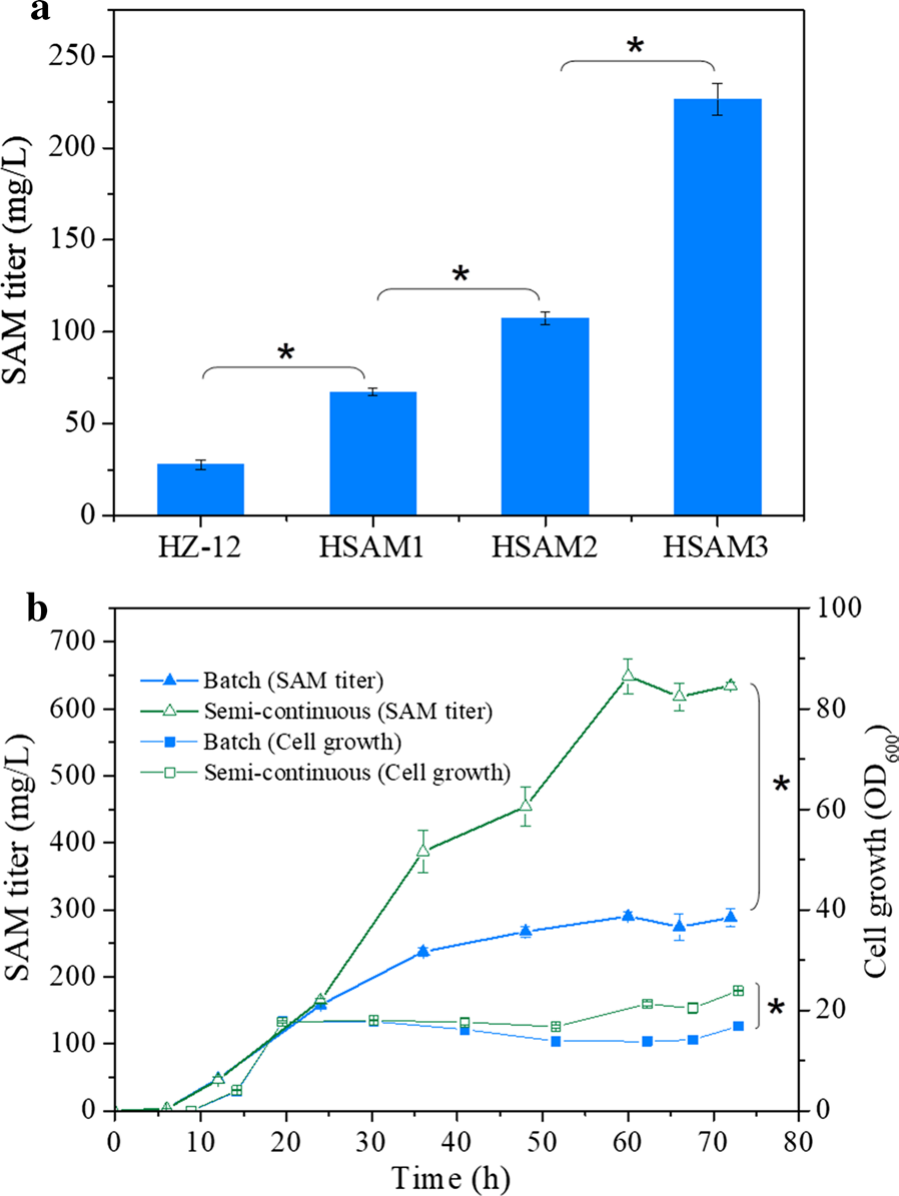
SAM synthesis of the strain HSAM3 in flasks. **a** SAM titer of HSAM3 compared with different strains in initial fermentation medium. **b** SAM titer and cell growth of HSAM3 under batch and semi-continuous batch fermentation based on the optimized fermentation medium. Data are expressed as mean ± SD from triplicate measurements. Asterisks indicate a significant difference at *p* < 0.05

To improve SAM production, plasmid PSAM2 carrying the most efficient *SAM2* gene was transformed into HSAM2 to generate HSAM3. Then, SAM titers of four key strains (HZ-12, HSAM1, HSAM2 and HSAM3) were compared under initial fermentation medium. Therein, the strain HSAM3 produced the maximum SAM titer of 226.61 mg/L, much higher than other strains (Fig. 5a). To reduce fermentation cost of HSAM3, the medium was optimized by adjustment of glucose and aspartate. Removing the glucose showed no significant effect on SAM titer, while further eliminating the aspartate significantly improved the SAM titer (Additional file 1: Fig. S4). Using optimized fermentation medium without glucose and aspartate, the maximum SAM titer reached 290.35 mg/L in 250 mL flasks containing 25 mL medium (Fig. 5b) and it was further improved to 648.99 mg/L by adding 2.5 mL of 10× optimized fermentation medium at 24 h and 48 h (semi-continuous batch fermentation), which was partly due to the increased cell growth (Fig. 5b). The batch and semi-continuous batch fermentation were also performed in a 3 L bioreactor, while the maximum SAM titer reached 242.01 mg/L and 424.21 mg/L, respectively (Additional file 1: Fig. S5). *B. amyloliquefaciens* can produce multiple surfactants, which led to the generation of excess foam in the 3 L bioreactor. Therefore, defoamer was added and the agitation speed was maintained at 400 rpm to avoid foam escaping from the bioreactor, which probably hindered SAM production.

This study aimed to improve SAM production using methionine-free medium. Therefore, previously reported SAM production results using methionine-free medium were compared at the flask scale (Table 1). The titer (290.35 mg/L), yield (30.28 mg/g DCW) and productivity (4.84 mg/L h) of HSAM3 in flask batch fermentation were much higher than previously reported [13, 15, 18]. Furthermore, the titer, yield and productivity were further improved by semi-continuous batch fermentation, demonstrating potential industrial applicability.

**Table 1:** Comparison of SAM production in this study with previous reports in methionine-free medium

Strain	Titer (mg/L)	Yield (mg/g DCW)	Productivity (mg/L h)	Medium carbon/nitrogen source (g/L)
*B. amyloliquefaciens* HSAM3	290.35	30.28	4.84	Sucrose 40, peptone 10, beef extract 5, urea 2, (NH_4_)_2_SO_4_ 6.3, flask batch fermentation
*B. amyloliquefaciens* HSAM3	648.99	46.42	10.82	Sucrose 120, peptone 30, beef extract 15, urea 6, (NH_4_)_2_SO_4_ 18.9, flask semi-continuous batch fermentation
*E. coli* Anti-argB [15, 18]	1.21	0.13	0.10	Glucose 20, NH_4_Cl 0.5, flask batch fermentation
*E. coli* SSP-1 [13, 18]	5.30	Not available	0.44	Glucose 20, NH_4_Cl 0.5, flask batch fermentation
*C. glutamicum* HW104/pJYW-4-*metK*-*vgb* [15]	196.7	12.15	4.10	Glucose 100, corn steep liquor 20, (NH_4_)_2_SO_4_ 20, flask batch fermentation

## Conclusions

This study presents an efficient strategy for enhancing SAM production by coupling an engineered SAM synthesis pathway and the TCA cycle. Recombinant plasmid-based expression of *SAM2* from *S. cerevisiae* and *metA* and *metB* from *E. coli*, in combination with deletion of *mccA*, resulted in significantly enhanced SAM production. Combining these genetic manipulations achieved a maximum SAM titer of 67.41 mg/L using the resulted strain HSAM1, a 1.42-fold increase compared with strain HZ-12. Transcription and metabolite analyses confirmed that the SAM synthesis pathway was strengthened. Unlike *C. glutamicum* using tetrahydropicolinate succinylase to drive succinyl-CoA into the lysine pathway, *B. amyloliquefaciens* just harbored homoserine O-succinyltransferase to mediate succinyl-CoA into the SAM pathway. Therefore, deleting the *sucC* gene forced the succinyl-CoA into the engineered SAM pathway, which was consequently coupled with the TCA cycle to generate a 0.59-fold increase of the SAM titer. Transcription and metabolite analyses also confirmed that pathway coupling diverted the metabolic flux toward the SAM pathway. Addition of plasmid-based expression of *SAM2* further increased the maximum SAM titer to 648.99 mg/L in methionine-free medium, much higher than previous reports. Thus, we developed an efficient strategy for enhanced SAM production, and provide a potential SAM production workhorse in the form of engineered *B. amyloliquefaciens*.

## Methods

### Strains and plasmids

All strains and plasmids used in this study are listed in Table 2. All resulted *B. amyloliquefaciens* strains were derived from the wild-type strain *B. amyloliquefaciens* HZ-12. *E. coli* DH5α was used to construct plasmids and amplify *metA*, *metB*, *thrA* and *metL*, and *Saccharomyces* *cerevisiae* CICC 31001 was used for amplification of *SAM2* and *YML082* *W* genes. Primers used in this study are included in Additional file 1: Table S1.

**Table 2:** Strains and plasmids used in this study

Strains or plasmids	Characteristics	Source
*B. amyloliquefaciens* strains		
HZ-12	CCTCC M2015234, wild type	Stored in lab
HZ-12 (PBmetK)	HZ-12 harboring the plasmid PBmetK	This study
HZ-12 (PSAM2)	HZ-12 harboring the plasmid PSAM2	This study
HZ-12 (PBmetA)	HZ-12 harboring the plasmid PBmetA	This study
HZ-12 (PEmetA)	HZ-12 harboring the plasmid PEmetA	This study
HZ-12 (PBmetI)	HZ-12 harboring the plasmid PBmetI	This study
HZ-12 (PEmetB)	HZ-12 harboring the plasmid PEmetB	This study
HZ-12 (PYML)	HZ-12 harboring the plasmid PYML	This study
HZ-12ΔmccA	HZ-12 deficient in *mccA*	This study
HZ-12:SAM2	HZ-12 integrated with SAM2 from *S. cerevisiae*	This study
HZ-12:SAM2(PthrA)	HZ-12:SAM2 harboring the plasmid PthrA	This study
HZ-12:SAM2(PmetL)	HZ-12:SAM2 harboring the plasmid PmetL	This study
HZ-12ΔmetEATAT	HZ-12 deficient in anti-antiterminator of *metE*	This study
HZ-12ΔsucC	HSAM1 deficient in *sucC*	This study
HSAM1	HZ-12 integrated with *SAM2*, *metA* and *metB*, and deficient in *mccA*	This study
HSAM2	HSAM1 deficient in *sucC*	This study
HSAM3	HSAM2 harboring the plasmid PSAM2	This study
*E. coli* DH5α	F^−^ Φ80d/*lac*ZΔM15, Δ(*lacZYA*-*argF*) U169, *recA*1, *endA*1, *hsdR*17 (*r*_K_^−^, *m*_K_^+^), *phoA*, *supE*44, λ^−^, *thi*-1, *gyrA*96, *relA*1	Stored in lab
*S. cerevisiae*	CICC 31001, wild type	Stored in lab
*B. subtilis* 168	Strain containing the P43 promoter	Stored in lab
*B. licheniformis* WX-02	CCTCC M208065, wild type	Stored in lab
Plasmids		
pHY300PLK	*E. coli*–*Bacillus* shuttle vector for gene expression, Ap^r^, Tet^r^	Stored in lab
PBmetK	pHY300PLK + P43 + TamyL + *metK* from HZ-12	This study
PSAM2	pHY300PLK + P43 + TamyL + *SAM2* from *S. cerevisiae*	This study
PBmetA	pHY300PLK + P43 + TamyL + *metA* from HZ-12	This study
PEmetA	pHY300PLK + P43 + TamyL + *metA* from *E. coli* DH5α	This study
PBmetI	pHY300PLK + P43 + TamyL + *metI* from HZ-12	This study
PEmetB	pHY300PLK + P43 + TamyL + *metB* from *E. coli* DH5α	This study
PYML	pHY300PLK + P43 + TamyL +* YML082* *W* from *S. cerevisiae*	This study
PthrA	pHY300PLK + P43 + TamyL + *thrA* from *E. coli* DH5α	This study
PmetL	pHY300PLK + P43 + TamyL + *metL* from *E. coli* DH5α	This study
T2(2)-ori	*E. coli*–*Bacillus* shuttle vector for gene knockout or integration; Kan^r^	Stored in lab
T2Δ*mccA*	T2 (2)-mccA (A + B) to delete *mccA*	This study
T2Δ*metE*ATAT	T2 (2)-metEATAT (A + B) to delete anti-antiterminator of *metE*	This study
T2Δ*sucC*	T2 (2)-sucC (A + B) to delete *sucC*	This study
T2-EmetA	T2 (2) + P43 + TamyL + *metA* from *E. coli* DH5α	This study
T2-EmetB	T2 (2) + P43 + TamyL + *metA* from *E. coli* DH5α	This study
T2-SAM2	T2 (2) + P43 + TamyL + *SAM2* from *S. cerevisiae*	This study

### Medium and culture conditions

Cells were inoculated into LB liquid medium (10 g/L tryptone, 5 g/L yeast extract, 10 g/L NaCl), and cultured for 12 h at 37 °C with shaking at 200 rpm to generate seed cultures. Seed cultures (inoculum size 3%) were then transferred into the fermentation medium. The 250 mL flask experiment was performed with 25 mL medium at 37 °C and 200 rpm. The 3 L bioreactor experiment was carried out in 1.2 L medium at 37 °C with an agitation speed of 400 rpm and an aeration ratio of 1.6 vvm. The initial fermentation medium comprised 10 g/L glucose, 40 g/L sucrose, 3 g/L aspartate, 10 g/L peptone, 5 g/L beef extract, 2 g/L urea, 6.3 g/L (NH_4_)_2_SO_4_, 2.5 g/L NaCl, 3 g/L KH_2_PO_4_ and 4.2 g/L MgSO_4_·7H_2_O (pH 6.5), which was used to investigate the effect of different genetic manipulation on SAM titer. For the final strain HSAM3, the initial fermentation was further optimized, and the optimized fermentation medium consisted of 40 g/L sucrose, 10 g/L peptone, 5 g/L beef extract, 2 g/L urea, 6.3 g/L (NH_4_)_2_SO_4_, 2.5 g/L NaCl, 3 g/L KH_2_PO_4_ and 4.2 g/L MgSO_4_·7H_2_O. Semi-continuous batch fermentation of HSAM3 was performed in optimized fermentation medium by repeated feeding 1/10 volume of 10× optimized fermentation medium at 24 h and 48 h.

### Recombinant plasmid expression

Recombinant plasmid expression was carried out based on the procedures reported previously [40, 41]. The *metK* gene was amplified from *B. amyloliquefaciens* HZ-12 using primers *metK*-F and *metK*-R, and the gene fragment was fused with the P43 promoter amplified from *B. subtilis* 168 and the TamyL terminator from *B. licheniformis* WX-02 by splicing with overlapping extension PCR (SOE-PCR) to generate the gene expression module. This was subsequently inserted into the pHY-300PLK plasmid between the *Bam*HI and *Xba*I restriction sites, resulting in recombinant expression plasmid PBmetK, which was electrotransformed into *B. amyloliquefaciens* competent cells to generate recombination strain HZ-12 (PBmetK). Other genes were expressed using the pHY-300PLK plasmid following the same procedure.

### Homologous recombination

Gene deletion and integration expression were performed by T2(2)-ori-mediated homologous recombination [40, 41]. To delete the *mccA* gene, primers Δ*mccA*-A-F/Δ*mccA*-A-R and Δ*mccA*-B-F/Δ*mccA*-B-R were designed to amplify two homologous arms (A and B) surrounding the *mccA* gene (~ 500 bp), which were further fused by SOE-PCR using primers Δ*mccA*-A-F/Δ*mccA*-B-R. The fused fragment was inserted into T2(2)-ori between the *Xba*I and *Bam*HI sites, resulting in knockout plasmid T2Δ*mccA*, which was electrotransformed into competent *B. amyloliquefaciens* cells. Cells were spread onto kanamycin-containing LB plates (20 μg/mL). After PCR verification, positive clones were inoculated into LB liquid medium containing kanamycin (20 μg/mL) and cultured for 8 h at 45 °C with shaking at 180 rpm. After further kanamycin resistance screening and PCR verification, single-crossover strains were picked, transferred into LB liquid medium, and sub-cultured at 37 °C (8 h) several times. Finally, subcultures were spread onto LB plates, and each single colony was streaked onto LB and kanamycin-containing LB plates, respectively, to select kanamycin-sensitive colonies. After further PCR verification, the double-crossover strain HZ-12Δ*mccA* was obtained. Deletion of *sucC* was carried out by the same method. For integrant expression, the *SAM2* gene expression module was fused with two homologous arms amplified from genomic prophage regions by SOE-PCR, and the fragment was inserted into the T2(2)-ori to generate the integrant expression vector T2-*SAM2*. The subsequent protocol was performed as described above for gene deletion.

### Detection of cell growth

The cell growth was monitored by measuring the OD_600_. At preset time, aliquots of culture broth were sampled and centrifuged at 12,000×*g* for 10 min to collect cell pellets. Then, the cell pellets were resuspended with distilled water and separated by centrifugation, which was repeated for three times to remove the residual medium. At last, the cells were resuspended to measure the OD_600_.

### Determination of SAM concentration

After fermentation, a 500 μL broth sample containing cells and medium was mixed with 1.5 mL perchloric acid solution (0.4 M), agitated by vortexing for 10 s every 15 min to extract intracellular and extracellular SAM for 1 h, and mixtures were centrifuged at 12,000×*g* for 10 min to collect the supernatant. Next, 800 μL supernatant, 95 μL NaOH (2 M) and 15 μL saturated NaHCO_3_ were mixed and centrifuged at 2500×*g* for 5 min. The supernatant was further filtered through a 0.22 μm membrane and the filtrate was subjected to SAM analysis by high-performance liquid chromatography (HPLC) [31]. Analysis was performed using a Zorbax Eclipse XDB-C18 column (4.6 mm × 250 mm, 5 μm) at 30 °C on an Agilent 1260 HPLC system (Agilent, USA). Methanol and 40 mM NH_4_H_2_PO_2_ solution containing 2 mM sodium heptane sulfonate served as mobile phases A and B at a ratio of 18:82. The flow rate was 0.8 mL/min and the detection wavelength was 254 nm.

### Transcription level analysis

Transcription levels were measured based on the method reported previously [42]. Total RNA from *B. amyloliquefaciens* cells was extracted with TRIzol Reagent (Invitrogen, USA), and residual DNA was removed by DNase I enzyme (TaKaRa, Japan). The cDNA was amplified with a RevertAid First-Strand cDNA Synthesis Kit (Thermo, USA). The target gene was amplified using corresponding primers in a 20 μL reaction system containing 2 μL cDNA, 1 μL primers, 10 μL SYBR Select Master Mix, and 7 μL DEPC water, and real-time PCR was performed at 95 °C for 5 min followed by 40 cycles of 95 °C for 30 s, 60 °C for 30 s and 72 °C for 30 s. Fragment specificity was verified through melting curve analysis at 95 °C for 1 min and 60 °C for 1 min, and transcription data were normalized using the 16S rDNA gene of *B. amyloliquefaciens* as an internal reference.

### Metabolite analysis

Intracellular metabolites were measured by gas chromatography–mass spectrometry (GC–MS) [43]. The cell culture was cooled to 9 ± 2 °C using liquid nitrogen and cells were collected by centrifugation at 6080×*g* for 5 min at 4 °C. Cell pellets were washed twice with 2 mL cold 0.85% (wt/vol) NaCl solution, resuspended in 2 mL cold 75% (v/v) ethanol solution containing 10 μg/L phenethyl acetate as an internal standard, and then frozen in liquid nitrogen for 3 min. Frozen cells were thawed and vortexed for 5 min to extract intracellular metabolites, which were collected by centrifugation and lyophilized. The lyophilized extract was dissolved in 100 μL methoxyamine hydrochloride pyridine solution (20 mg/mL), oximated for 1 h at 37 °C, and derived with 100 μL *N*-methyl-*N*-(trimethylsilyl)-trifluoroacetamide for 3 h at 60 °C. Derivatives were determined using a TSQ8000 Evo gas chromatograph/mass spectrometer with a DB-5 MS capillary column (30 m × 0.25 μm × 250 μm) (Thermo Fisher, USA). The initial column temperature (50 °C) was ramped to 110 °C at 10 °C/min and held for 3 min, then raised to 165 °C at 2 °C/min, and finally to 220 °C at 3 °C/min and held for 10 min. The temperature of the MS transfer line and ion source was 280 °C and 300 °C, respectively. The scanned range was 50–650 *m/z*, and the helium carrier gas low rate was 1.2 mL/min. Aspartate, succinate, fumarate, homocysteine and methionine in samples were identified by comparing the *m/z* value, retention time, and fragmentation pattern with corresponding standards. Peak retention time alignment and peak area integration were carried out by Thermo TraceFinder 4.1 (Thermo Fisher, USA), and the peak area was normalized using an internal standard. The fold change of each metabolite was calculated according to their peak areas.

### Statistical analysis

All data were collected at least in triplicate to calculate the mean value and standard deviation, and significance *t* tests were used to determine differences at the 95% confidence level using Data Processing System (DPS) 7.05.

## Supplementary information

**Additional file 1.** Additional tables and figures.

## Data Availability

Not applicable.
