# Supplementary material for: Metabolic engineering of Bacillus amyloliquefaciens for enhanced production of S-adenosylmethionine by coupling of an engineered S-adenosylmethionine pathway and the tricarboxylic acid cycle
Source: Biotechnol Biofuels. 2019 Sep 9;12:211. doi: 10.1186/s13068-019-1554-0 (PMC6732833; doi:10.1186/s13068-019-1554-0)
Supplement: Supplementary file 1 — Additional file 1. Additional tables and figures. [file 13068_2019_1554_MOESM1_ESM.docx]

**Additional materials**

**Metabolic engineering of *Bacillus amyloliquefaciens* for enhanced production of** **S-adenosylmethionine by coupling of an engineered S-adenosylmethionine pathway and the tricarboxylic acid cycle**

Liying Ruan^a,1^, Lu Li^a,1^, Dian Zou^a^, Cong Jiang^a^, Zhiyou Wen^a,d^, Shouwen Chen^c^, Yu Deng^b*^, Xuetuan Wei^a*^

^a^ *Key Laboratory of Environment Correlative Dietology (Ministry of Education),* *College of Food Science and Technology,* *Huazhong Agricultural University, Wuhan 430070, China*

^b^ *National Engineering Laboratory for Cereal Fermentation Technology (NELCF), Jiangnan University, 1800 Lihu Road, Wuxi, Jiangsu 214122, China*

^c^ *Hubei Collaborative Innovation Center for Green Transformation of Bio-Resources，College of Life Sciences, Hubei University, Wuhan 430062, China*

^d^ *Department of Food Science and Human Nutrition, Iowa State University, 50011 Ames, IA, USA*

*Address Correspondence:

Xuetuan Wei: weixuetuan@mail.hzau.edu.cn. Tel: +86-27-87280670. Fax: +86-27-87280670.

Yu Deng: dengyu@jiangnan.edu.cn, Tel: +86-510-85329031; Fax: +86-510-85918309.

^1^These authors contributed equally to this work.

**Table S1 Primers for PCR**

| Primer name | Sequence of primer (5' to 3') |
| --- | --- |
| P43-F | TGATAGGTGGTATGTTTTCG |
| P43-R | TTCATGTGTACATTCCTCTC |
| Tamyl-F | AAGAGCAGAGAGGACGGATT |
| TamyL-R | CGCAATAATGCCGTCGCACT |
| *metK*-F | GAGAGGAATGTACACATGAATTATTCTCCTAGTGCTTCTTTGC |
| *metK*-R | AATCCGTCCTCTCTGCTCTTATGAGTAAAAACCGTCGACTATT |
| *SAM2-*F | GAGAGGAATGTACACATGAAATGTCCAAGAGCAAAACTTTC |
| *SAM2-*R | AATCCGTCCTCTCTGCTCTTTTAAAATTCCAATTTCTTTGGT |
| *metA*-F | GAGAGGAATGTACACATGAATTGCCTATTAATATACCAAATCACC |
| *metA*-R | AATCCGTCCTCTCTGCTCTTTTAATCCCATTCATACGGAGT |
| *EmetA*-F | GAGAGGAATGTACACATGAAATGCCGATTCGTGTGCCG |
| *EmetA*-R | AATCCGTCCTCTCTGCTCTTTTAATCCAGCGTTGGATTCA |
| *metI*-F | GAGAGGAATGTACACATGAAATGACGCAGCATGTTGAAAC |
| *metI*-R | AATCCGTCCTCTCTGCTCTTTTACTCATATGACACGGCCC |
| *metB*-F | GAGAGGAATGTACACATGAA ATGTCTGAGCCGATGATGTG |
| *metB*-R | AATCCGTCCTCTCTGCTCTTTTACTTGCGAGTGACAGCC |
| *YML*-F | GAGAGGAATGTACACATGAAATGGTTTCAGCGCAAGTG |
| *YML*-R | AATCCGTCCTCTCTGCTCTTTCATATTTCTACCGTTTTATCAAT |
| *thrA*-F | GAGAGGAATGTACACATGAAATGCGAGTGTTGAAGTTCGG |
| *thrA*-R | AATCCGTCCTCTCTGCTCTTTCAGACTCCTAACTTCCATGAG |
| *metL*-F | GAGAGGAATGTACACATGAAATGAGTGTGATTGCGCAGG |
| *metL*-R | AATCCGTCCTCTCTGCTCTTTTACAACAACTGTGCCAGCC |
| pHY300-F | GTTTATTATCCATACCCTTAC |
| pHY300-R | CAGATTTCGTGATGCTTGTC |
| Δ*mccA*-A-F | CG**GGATCC**GAGCTCTTTATCAGCCGTCAC |
| Δ*mccA*-A-R | CAGATATTACAGAACTGATCGGAAACATTTTTCAAGGAGGTCAG |
| Δ*mccA*-B-F | CTGACCTCCTTGAAAAATGTTTCCGATCAGTTCTGTAATATCTG |
| Δ*mccA*-B-R | GC**TCTAGA**TACGACGGGCAGCTATGAAG |
| Δ*mccA*-Y-F | TATCAAAGGAAATCATTCCGC |
| Δ*mccA*-Y-R | ATCATCAATTAGCGGACGAT |
| Δ*sucC*-A-F | CG**GGATCC**GACCCTTTTTATGAATGTATCC |
| Δ*sucC*-A-R | GAATATCCATGAGTACCAGGGAATCTGCGGAATCAATGGC |
| Δ*sucC*-B-F | GCCATTGATTCCGCAGATTCCCTGGTACTCATGGATATTC |
| Δ*sucC*-B-R | GC**TCTAGA**AGAAAGATCAAGCCCCGA |
| Δ*sucC*-Y-F | ATCTTCATTGAACGCTTTTAAC |
| Δ*sucC*-Y-R | CTTGTGGAAGAGTTTATAGAAAAAG |
| ΔmetEATAT-A-F | TCC**CCCGGG**CCGATAACAGCGGACAGAT |
| ΔmetEATAT-A-R | GCCTCTTATCTCTCAGATAACATCTTCGCCCAACTCTCGATAAG |
| ΔmetEATAT-B-F | CTTATCGAGAGTTGGGCGAAGATGTTATCTGAGAGATAAGAGGC |
| ΔmetEATAT-B-R | GC**TCTAGA**ACCGATATGAACCGATAAGTTC |
| ΔmetEATAT-Y-F | AAACATACAACGTACACCTCGT |
| ΔmetEATAT-Y-R | TTCGCATCAAGCGCATAAT |
| *SAM2*-A-F | CG**GGATCC**CTTCGGAACTGACACCGT |
| *SAM2*-A-R | CGAAAACATACCACCTATCAAAGCGCCAAAATCGTAAAC |
| *SAM2*-B-F | AGTGCGACGGCATTATTGCGGAAAATGGTCGGCGTGTT |
| *SAM2*-B-R | GC**TCTAGA**GGCGCAGTCAAGCAATTT |
| *SAM2*-Y-F | CGGAAAATGAACGAGAAAC |
| *SAM2*-Y-R | GAAATCGGGAATGCTGTGA |
| *EmetA*-A-F | CG**GGATCC**CGGGCTCAAGACTTCAGT |
| *EmetA*-A-R | CGAAAACATACCACCTATCACCTTTTTAGACGGTGAGCA |
| *EmetA*-B-F | AGTGCGACGGCATTATTGCGCATTACGCCCGTGTCTGT |
| *EmetA*-B-R | GC**TCTAGA**CGCCTTATCGACATAGCTC |
| *EmetA*-Y-F | AAGAGTCATACAATGCGGTC |
| *EmetA*-Y-R | GGCCTTTTCAATCTCTTCAG |
| *metB*-A-F | CG**GGATCC**ACCAGATCACGCCATACGAT |
| *metB*-A-R | CGAAAACATACCACCTATCATCAAGAAACAGCGCATCATC |
| *metB*-B-F | AGTGCGACGGCATTATTGCGGAGCTATGTCGATAAGGCGG |
| *metB*-B-R | GC**TCTAGA**TTCTCATCTTGTTCAAGTCCTCT |
| *metB*-Y-F | AGCGAGCTGGCGTAGTCAC |
| *metB*-Y-R | CTCTTCTTCCGGCTGCTT |
| T2-F | ATGTGATAACTCGGCGTA |
| T2-R | GCAAGCAGCAGATTACGC |
| *lysC*-RT-F | CCATCATCCGACTGTGTCTG |
| *lysC*-RT-R | GTCAAGCACGGAACATGAAG |
| *asd*-RT-F | GCCATCGTGATTGACAATACG |
| *asd*-RT-R | CAGCTTGGTAAGTAGATACAATGAC |
| *hom*-RT-F | TTGGAAGCGATTCGTATTG |
| *hom*-RT-R | CATCGATCACATCATAGACTTCTG |
| *metA*-RT-F | TACCAAATCACCTGCCCG |
| *metA*-RT-R | TGTGTGCTCGGAATTAAGAAC |
| *metI*-RT-F | ATGACGCAGCATGTTGAAAC |
| *metI*-RT-R | CCGAGCTGAAAGCAAATCC |
| *metC*-RT-F | CTGTCGAGTCCATTCTCTCATAC |
| *metC*-RT-R | TTATGATCTCACTTGGGCG |
| *metE*-RT-F | AGAGCTTTCAGGGCTGAATG |
| *metE*-RT-R | ATAACTCCTCAACATCAGCGTG |
| *metK*-RT-F | TTATTCTCCTAGTGCTTCTTTGC |
| *metK*-RT-R | AACTGATCGAAGTCGTACGG |
| *SAM2*-RT-F | CAAGGTTGCCTGTGAAACAG |
| *SAM2*-RT-R | CATAGTGCAGACCTTGAGCG |
| *EmetA*-RT-F | ATGATGTCGCTTACTGGCC |
| *EmetA*-RT-R | AATCATCAAAGCCACGCG |
| *metB*-RT-F | ATGTCTGAGCCGATGATGTG |
| *metB*-RT-R | GCAAGATAATGAACGGGATG |
| 16S-RT-F | GGATGTCAAGACCTGGTAAGG |
| 16S-RT-R | CGCCGTAAACGATGAGTG |

Note: Underline stands for the overlap region for Splicing by Overlapping Extension PCR (SOE-PCR).


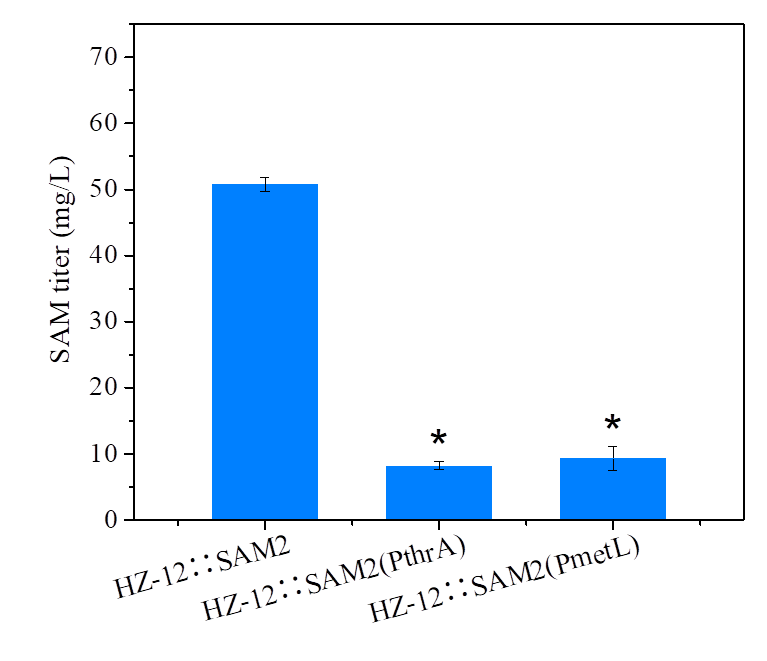


**Fig.S1. Effect of expression of bifunctional aspartokinase/homoserine dehydrogenase genes on SAM production.** HZ-12::SAM2: the HZ-12 strain integrated with SAM2 gene in genome; HZ-12::SAM2(PthrA): HZ-12::SAM2 harboring the plasmid carrying *thrA* gene from *E. coli*; HZ-12::SAM2(PmetL): HZ-12::SAM2 harboring the plasmid carrying *metL* gene from *E. coli*. Data are expressed as means ± SD of three replications. Asterisks indicate a significant  difference (P <0.05) compared with the control (HZ-12::SAM2).


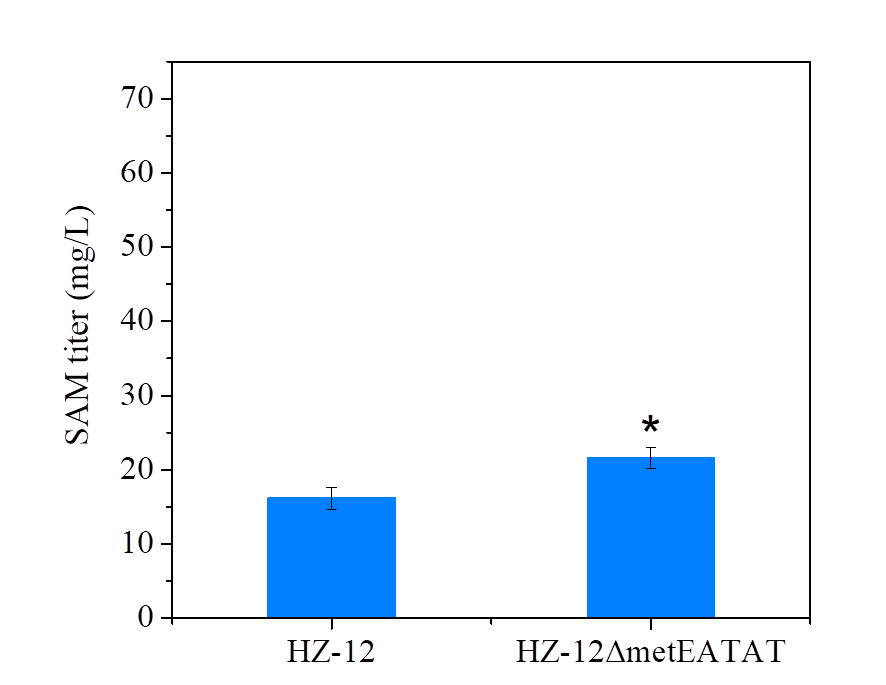


**Fig.S2. Effect of deletion of anti-antiterminator of *metE* gene on SAM production.** HZ-12ΔmetEATAT: the HZ-12 strain deficient in anti-antiterminator of *metE*. Data are expressed as means ± SD of three replications. Asterisk indicates a significant  difference (P <0.05) between HZ-12 and HZ-12ΔmetEATAT.


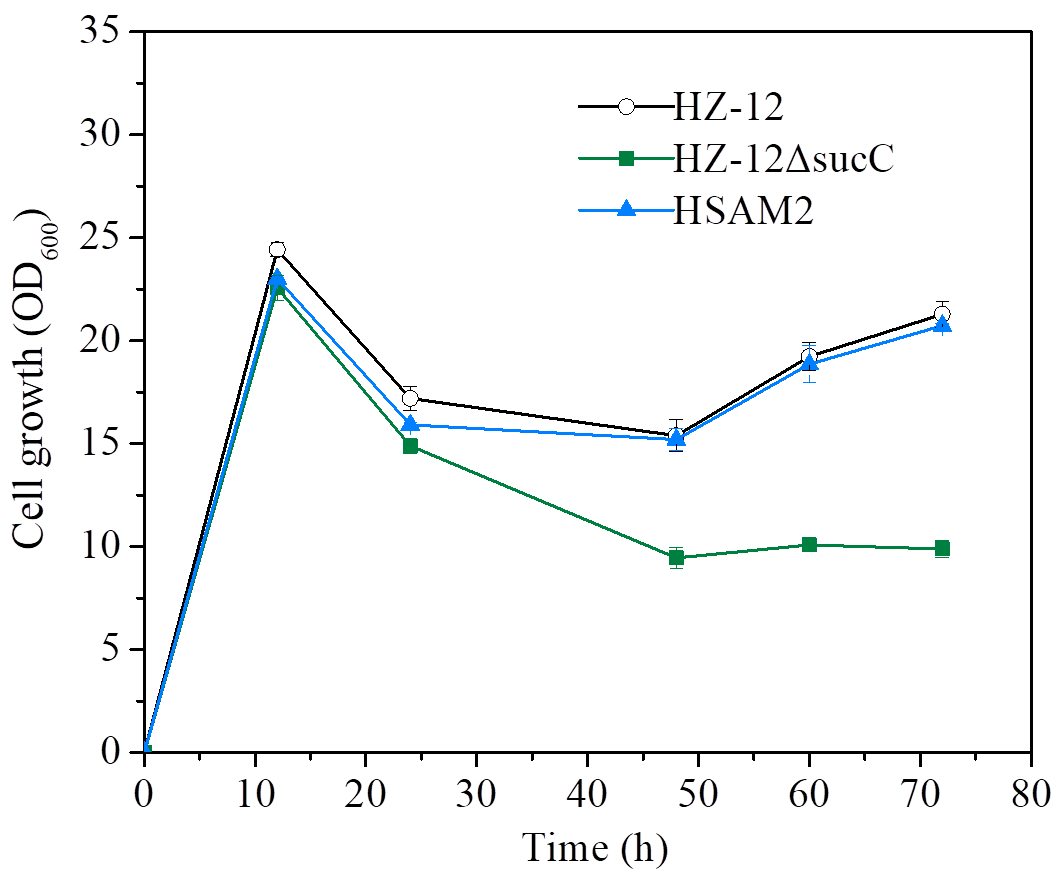


**Fig.S3. Cell growth for wild-type strain (HZ-12), *sucC*-deficient strain (HZ-12ΔsucC) and *sucC*-deficient strain harboring the engineered SAM pathway (HSAM2).** Data are expressed as means ± SD of three replications.


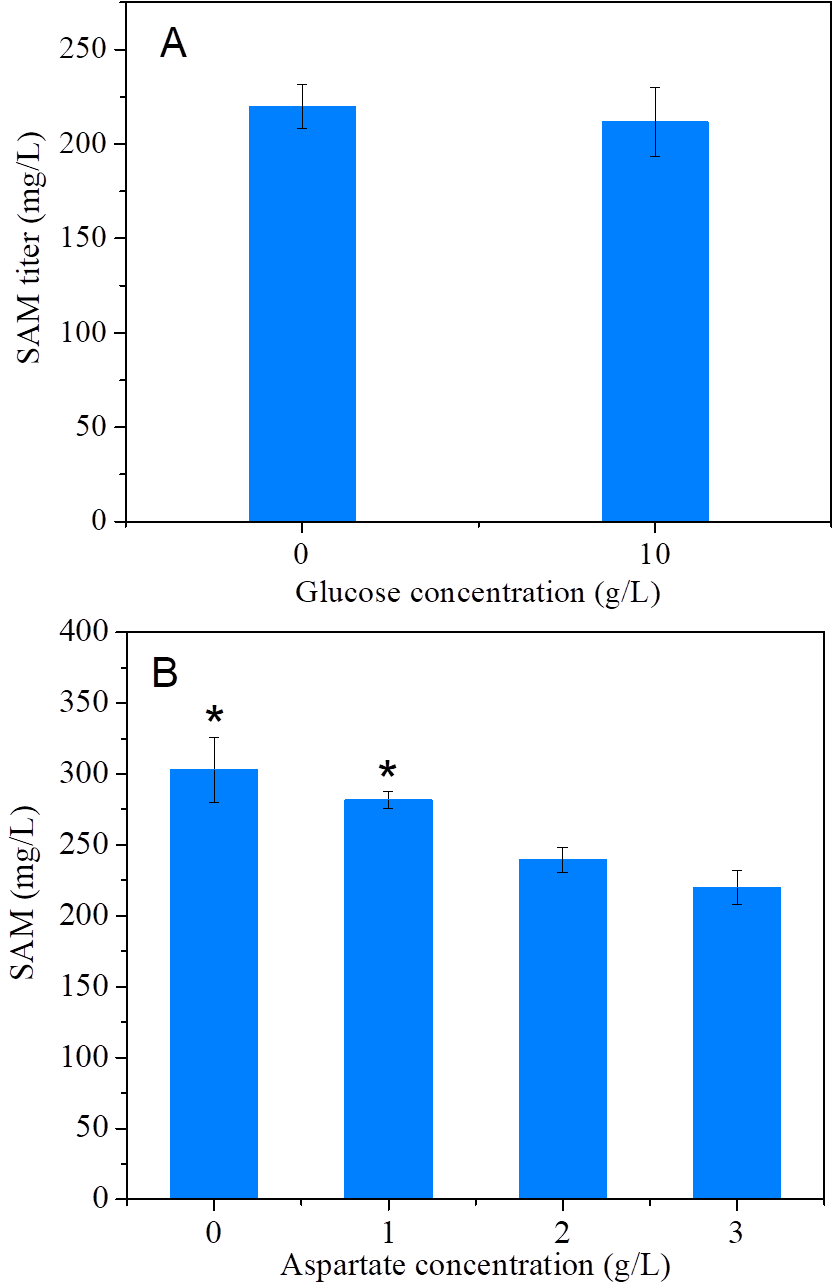


**Fig.S4. Effects of glucose and aspartate on SAM production.** (A) Effect of glucose. (B) Effect of aspartate. Data are expressed as means ± SD from triplicate measurements. Asterisks indicate the significant difference (*p* <0.05) of the SAM titers at 0 or 1 g/L aspartate compared with that of 3 g/L aspartate.

**
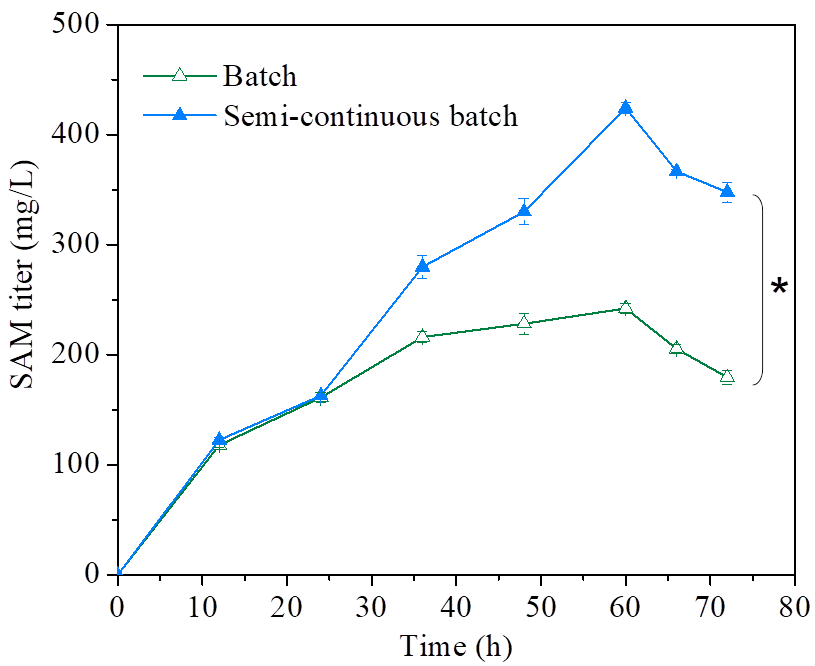
**

**Fig.S5. SAM synthesis by strain HSAM3 under batch and semi-continuous batch fermentation a 3 L bioreactor.** The batch and semi-continuous batch fermentation experiments were carried out based on the optimized fermentation medium. Data are expressed as means ± SD from triplicate measurements. Asterisks indicate a significant difference (*p* <0.05) between batch and semi-continuous batch fermentation.
